# A short-term longitudinal study of correlates and sequelae of attachment security in autism

**DOI:** 10.1080/14616734.2017.1383489

**Published:** 2017-09-29

**Authors:** Agata Rozga, Erik Hesse, Mary Main, Robbie Duschinsky, Leila Beckwith, Marian Sigman

**Affiliations:** ^a^ School of Interactive Computing, Georgia Institute of Technology, Atlanta, GA, USA; ^b^ Department of Psychology, University of California, Berkeley, CA, USA; ^c^ Department of Public Health and Primary Care, University of Cambridge, Cambridge, UK; ^d^ Department of Psychiatry, University of California, Los Angeles, CA, USA; ^e^ Department of Pediatrics, University of California, Los Angeles, CA, USA

**Keywords:** Attachment, autism, parent–child interaction, maternal sensitivity, empathy

## Abstract

In this short-term longitudinal study, 30 preschool-aged children with autism were first observed in Ainsworth’s Strange Situation Procedure and, separately, interacting with the primary caregiver in the home. One year later, each child completed both a developmental assessment and an observational assessment of empathic responding. Behaviors typical for children with autism were distinguished from behaviors suggestive of relationally based attachment disorganization. Forty-five percent of the children were classified as securely attached. The secure group demonstrated language skills superior to those of the insecurely attached group, concurrently and during the follow-up. Compared to parents of children who were insecurely attached, parents of securely attached children were rated as more sensitive. Compared to both organized insecure and disorganized children, secure children were rated as more responsive to an examiner’s apparent distress during the follow-up relative to their ratings at intake, whereas empathy ratings of children with insecure classifications did not increase. Importantly, attachment security was associated with empathy above and beyond the contribution of children’s language level. These results indicate that the sequelae of attachment security in autism may be similar to those documented for typically developing children.

In contrast to early clinical observations that characterized autism as involving a failure to develop an expectable attachment (American Psychiatric Association, 1987; Cohen, Paul, & Volkmar, 1987; Kanner, 1943; Rutter, 1978), subsequent empirical investigations have indicated that children with autism ordinarily develop attachments to their primary caregivers and, in fact, that a sizeable subgroup form secure attachments (for a meta-analytic review, see Rutgers, Bakermans-Kranenburg, Van IJzendoorn, & Van Berckelaer-Onnes, 2004). A principal task facing researchers today is to shed light on the processes through which children with autism forge secure attachments, given their well-documented differences in intersubjective understanding and social motivation relative to neurotypical children of comparable age (e.g. Mundy, Sigman, Ungerer, & Sherman, 1986; Nielsen, Slaughter, & Dissanayake, 2013; Oberman et al., 2005; Sigman & Capps, 1997). Though there is an emerging literature on the social and cognitive correlates of attachment in autism (e.g. Capps, Sigman, & Mundy, 1994; Koren-Karie Oppenheim, Dolev, & Yirmiya, 2009, 2007b; Naber et al., 2007a; Oppenheim, Koren-Karie, Dolev & Yirmiya, 2009; Van IJzendoorn et al., 2007; Willemsen-Swinkles, Bakermans-Kranenburg, Buitelaar, van IJzendoorn, & van Engeland, 2000), the implications of secure attachment for subsequent development in children with autism remains an understudied topic (Kahane & El-Tahir, 2015). To that end, the goals of the present study were to (1) investigate the correlates of individual differences in the quality of attachment organization in children with autism, including maternal sensitivity as well as child cognitive and social skills, and, (2) for the first time, examine the implications of attachment security for subsequent development of these children. With respect to the latter, we focus on children’s response to another person’s apparent distress during a procedure administered 1 year after the attachment assessment.

## Attachment organization in autism

Early studies demonstrated that 2–5-year-old children with autism show discriminative attachment responses, including social behaviors such as looks, touches, and vocalizations directed preferentially toward their caregiver over a stranger, and proximity-seeking of the caregiver upon reunion following a brief separation (Rogers, Ozonoff, & Maslin-Cole, 1991, 1993; Shapiro, Sherman, Calamari, & Koch, 1987). Subsequently, Dissanayake and Crossley (1996, 1997) documented that preschool-age children with autism rely on their caregiver as a secure base for exploring their environment and a haven of safety in times of alarm, phenomena which are considered central in indicating the presence of attachment (Ainsworth, 1973). These findings have been extended to toddlers with autism, who have shown distress and search behaviors upon separation from the parent (Esposito, Rostagno, Venuti, Haltigan, & Messinger, 2014).

In addition to attachment-related behaviors per se, there are expectable individual differences in the overall quality of the attachment relationship which children with autism form with their caregivers. Studies using the Ainsworth Strange Situation procedure (Ainsworth & Wittig, 1969) find that 40–60% of preschool-age children with autism are classified as securely attached, based on Mary Ainsworth’s original secure/avoidant/resistant classification system (Capps et al., 1994; Koren-Karie et al., 2009; Shapiro et al., 1987; Van IJzendoorn et al., 2007; Willemsen-Swinkles et al., 2000). In the first Strange Situation study conducted with toddlers, Naber et al. (2007a) found that 35% of toddlers with a diagnosis of Autistic Disorder and 43% of toddlers with a diagnosis of Pervasive Developmental Disorder Not Otherwise Specified (PDD-NOS) were classified as securely attached (see also Naber et al., 2008).

One challenge in assessing attachment security in autism is differentiating the stereotyped and repetitive behaviors that these children often exhibit from those signifying relationally based disorganization (Pipp-Siegel, Siegel, & Dean, 1999). These behaviors only become codable as indices of disorganized attachment when they are either not explicable in other terms (e.g. neurological) or they are only readily explicable as stemming from a conflict associated with the child being alarmed by the caregiver (Hesse & Main, 2006; Main & Hesse, 1990). Only in this latter case can the behavior be understood to reflect conflict or disruption at the level of the attachment behavioral system.

In the first study of attachment in autism to include the D classification, all children whose attachment status could be classified with the Ainsworth tripartite system additionally showed behaviors listed in the Main and Solomon indices (Capps et al., 1994). Six (40%) of the children were classified as alternate secure, and this group was unique in that – apart from the repetitive hand and eye movements, odd facial movements, and rocking and circling behavior often observed in children with autism – they showed no signs of disorganization. These findings suggest that these behaviors were not indicative of a relationally based disorganization for these children, who should therefore be regarded as securely attached when coding this population. In support of this conclusion, the mothers of the six children who were classified as alternate secure were concurrently rated as more sensitive than mothers of the children classified as alternate insecure, as has been reported of the caregivers of secure infants in typically developing samples (see e.g. Ainsworth, Blehar, Waters, & Wall, 1978; Pipp-Siegel et al., 1999).

Some preliminary empirical evidence has already indicated that attachment disorganization can be validly differentiated from autistic stereotypies often observed in children with autism. Willemsen-Swinkels and colleagues (2000) first assigned children a secure/avoidant/resistant classification, and then additionally classified each child as disorganized or not, with stereotyped behaviors excluded from consideration. Children with autism classified as disorganized in this way had greater average heart rate changes during the separation and reunion episode than those who were not assigned the D classification. These findings were subsequently replicated in a toddler sample by Naber et al. (2007a). These findings suggest that when autistic stereotypies are excluded from consideration, the disorganized attachment classification can nonetheless be assigned for children with autism, with expectable correlates (see also Koren-Karie et al., 2009; Van IJzendoorn et al., 2007).

## Maternal behavior and child abilities as correlates of attachment in autism

For children with autism, different patterns of behavior in the Strange Situation have been linked to expectable differences observed in interactions with their mothers. As has been demonstrated for the mothers of typically developing children, mothers of securely attached preschoolers with autism have been rated as more sensitively responsive than mothers of insecurely attached children (Capps et al., 1994; Koren-Karie et al., 2009). One study did not find a link between sensitivity and attachment security in a sample of toddlers with autism (Van IJzendoorn et al., 2007). This study utilized a median split on maternal sensitivity and compared continuous ratings of child attachment security between mothers rated as high versus low in sensitivity (rather than comparing continuous ratings of maternal sensitivity between securely vs. insecurely attached children, as the other studies had done). Koren-Karie and colleagues (2009) reported that the relationship between maternal sensitivity and attachment security in autism holds after controlling for children’s level of developmental functioning and responsiveness to mother during a separate interaction. In the study perhaps most clearly corroborating this sensitivity–security link, Siller, Swanson, Gerber, Hutman, and Sigman (2014) reported that an intervention aimed at improving caregiver sensitivity with children with autism led (post-intervention) to an increase in attachment behaviors exhibited by the children during a modified separation and reunion procedure.

Oppenheim and colleagues (2009) have identified a parent’s insightfulness into their child’s experience and the extent to which they have come to terms with their child’s diagnosis as variables that increase the likelihood of secure attachment in children with autism, as has been demonstrated for typically developing children (Koren-Karie, Oppenheim, Dolev, Sher, & Etzion-Carasso, 2002) and children with medical diagnoses (Marvin & Pianta, 1996). In the Oppenheim, Koren-Karie, Dolev, and Yirmiya (2012) study, the link between maternal insightfulness and attachment security in autism was found to be mediated by maternal sensitivity, suggesting that parental appreciation of the child’s apparent subjective experience forms one important basis for sensitive behavior in mothers of children with autism.

A final consideration in examining links between offspring attachment security and maternal sensitivity in autism concerns the apparent prevalence of disorganized attachment. As implied by this review, the organized forms of insecurity (i.e. avoidant and resistant) and disorganization are often independent. Thus, it is quite common to observe infants who not only use their caregiver primarily as a safe haven in the Strange Situation but also display some conflict or anomalous behavior in the course of doing so (i.e. disorganized/secure; D/B). Main and Hesse (1992) note that such behavior can emerge when a caregiver not only is predominantly sensitive and responsive to their child’s attachment signals but also occasionally engages in displays of behavior which alarms the child (including not only directly frightening but also frightened and dissociative behavior [FR]; see Hesse & Main, 1999, 2006). In support of this account, in the results of a meta-analysis of precursors, concomitants, and sequelae of disorganized attachment, Van IJzendoorn, Schuengel, and Bakermans-Kranenburg (1999) indicated no significant relationship between disorganized attachment in infants and ratings of maternal sensitivity, as assessed using Ainsworth’s sensitivity–insensitivity scale.

While the above findings lend support for the role of maternal sensitivity in fostering secure attachment in autism, it is also the case that some of the studies cited above found significant associations between attachment security and various aspects of child cognitive skill and social-communication abilities. Compared to children with autism judged as insecurely attached, children with autism judged as securely attached have been found to have better receptive language (Capps et al., 1994) and play skills (Naber et al., 2008), and to make more social initiations during play with their caregivers (Capps et al., 1994; Willemsen-Swinkels et al., 2000). In addition, they have been found to be more responsive to an examiner’s bids for joint attention and to direct more requesting bids to her during a standardized assessment (Capps et al., 1994). Links between severity of autism symptoms and attachment security have been equivocal. Studies utilizing total scores on the Autism Diagnostic Observation Schedule (ADOS) diagnostic assessment reported significant differences in total scores between securely and insecurely attached children (Naber et al., 2007a; Van IJzendoorn et al., 2007). In contrast, studies comparing children with strictly defined autism to children with diagnoses of PDD-NOS reported similar rates of security in the two groups (Oppenheim et al., 2009; Willemsen-Swinkels et al., 2000). Finally, disorganization appears specifically linked with intellectual disability in autism (Koren-Karie et al., 2009; Naber et al., 2007a; Van IJzendoorn et al., 2007; Willemsen-Swinkels et al., 2000). However, these findings are correlational and cross-sectional, and further longitudinal research is needed to clarify the exact meaning of these associations.

In their meta-analysis of parental antecedents of infant attachment, De Wolff and Van IJzendoorn (1997) found a moderate association (effect size of *r* = .24) between sensitivity, as measured using the Ainsworth scales, and secure attachment, as measured via the Strange Situation. Several other domains of maternal interactive behavior, including mutuality and synchrony, showed similar effect sizes. The association between maternal behavior and infant attachment was significantly weaker in studies of clinical samples. Thus, while this meta-analysis confirmed the role that sensitivity plays in the development of infant attachment security, it also indicated that sensitivity may not capture the only mechanism through which the development of secure attachment is shaped. Additional influences impacting attachment may be relevant, especially for clinical populations. As others have argued, caregiver sensitivity must be considered within the context of the interactive processes of the parent–infant relationship, including the contribution of child characteristics to the attachment relationship (Belsky, 1997). Among the studies of maternal sensitivity and attachment in autism cited above, few have systematically examined the impact of alterations in children’s social, cognitive, and language skills on the quality of attachment in autism. Moreover, because these studies measured child characteristics and maternal sensitivity at the same time, they cannot address the obvious question of causality, namely whether observed child behaviors are antecedents or outcomes of sensitive parenting in autism.

## Developmental consequences of attachment security in autism

Research on attachment in children with autism summarized in previous sections indicates that a large proportion of children with autism develop secure attachments and that individual differences in the quality of attachment can be linked to aspects of interactions with caregivers. Empirical studies are now needed to investigate whether the secure attachments assessed in this population predict subsequent socio-emotional functioning in children with autism, as has been demonstrated for typically developing children.

Research with typically developing children has highlighted the greater interpersonal competence of those with secure attachments. Children with secure classifications have been rated as more socially competent, empathic, and popular with their peers in preschool and early childhood than children with insecure classifications (Belsky & Fearon, 2002; Grossmann & Grossmann, 1991; Shulman, Elicker, & Sroufe, 1994; Sroufe, 1983, 2005; Thompson, 2008). Increased empathic responsiveness on the part of children with secure attachment histories relative to those with anxious and avoidant histories has been documented even during infancy (Main & Weston, 1981), as well as preschool teacher ratings (e.g. Weinfield, Sroufe, Egeland, & Carlson, 1999) and direct observations of empathic behavior with peers on the playground, specifically responses to another child’s distress (e.g. Kestenbaum, Farber, & Sroufe, 1989; Sroufe, 1983).

In the current study, we assessed congruent changes in children’s affect and looking time to an examiner while she expressed apparent distress upon “hurting” her finger while playing with a toy. This paradigm had been previously shown to elicit such empathic responses from typically developing infants (Zahn-Waxler, Robinson, & Emde, 1992; Zahn-Waxler & Radke-Yarrow, 1982) as well as children with autism and with intellectual disability (Dawson et al., 2004; Sigman, Kasari, Kwon, & Yirmiya, 1992). Such early-emerging behavioral responses to another person’s distress have been found to be developmentally linked with later expressions of empathy (Hutman & Dapretto, 2009; Zahn-Waxler, Radke-Yarrow, Wagner, & Chapman, 1992) and thus represent a good proximal domain for an initial exploration of the developmental consequences of attachment security in autism. A focus on empathy is further motivated by previous studies indicating that preschool-age children with autism (Dawson et al., 2004; Sigman et al., 1992) as well as infants and toddlers (Charman et al., 1997; Hutman et al., 2010) are less attuned to the displays of distress of others and are less likely to engage in comforting behaviors when compared to typically developing children.

## Current study

The present short-term *longitudinal* study extends the existing body of research by examining the correlates and, for the first time, *sequelae* of attachment security (B) in children with autism, compared to children who are classified as organized insecure (A or C) or disorganized (D).

First, we examined the continuity between quality of attachment and later empathic responding. Based on documented links in the literature examining empathic response in typically developing children (e.g. Kestenbaum et al., 1989; Main & Weston, 1981; Sroufe, 1983), we hypothesized that compared to children classified as organized insecure (A and C) and disorganized (D), children with autism classified as securely attached (B) would be rated as more empathically responsive to another person’s distress during a 1-year follow-up.

Second, we examined the cognitive–linguistic correlates of attachment security in children with autism, concurrently and during a 1-year follow-up. Attachment theory posits that the influence of attachment relationships should be particularly apparent in the domains of emotion regulation and interpersonal closeness. However, associations between individual differences in quality of attachment and symbolic and cognitive abilities have been reported among samples of children with autism (e.g. Naber et al., 2008; Willemsen-Swinkels et al., 2000), typically developing children (see Van IJzendoorn, Dijkstra, & Bus, 1995 for a meta-analysis) and children with Down syndrome (Atkinson et al., 1999). We hypothesized that, compared to children with autism classified as organized insecure or disorganized, children classified as securely attached would have better language skills and that these group differences would persist through the 1-year follow-up.

Finally, we examined the role of individual differences in caregiver sensitivity in the development of attachment in autism, building in part upon the few existing studies examining this link and to shed light on inconsistencies in this prior work (Capps et al., 1994; Koren-Karie et al., 2009; Oppenheim et al., 2012; Van IJzendoorn et al., 2007). We examined relationships between attachment security as assessed in the laboratory and parents’ and children’s behavior in a *separate interaction in the home*, and relationships between attachment security and maternal sensitivity both with and without consideration of disorganization. We considered potential differences between (1) children with autism classified as secure versus insecure, relying only on Ainsworth classifications (i.e. the B vs. A or C comparison) and excluding the additional information regarding disorganization (D), and (2) differences between children classified as secure versus children classified as organized insecure (A or C) and as disorganized (D). From a theoretical perspective (Out, Bakermans-Kranenburg, & Van IJzendoorn, 2009), we expected the former contrast to show a stronger association with caregiver sensitivity. Second, considering prior studies indicating that children with autism whose attachments are judged as insecure direct fewer social bids to their caregivers (Capps et al., 1994; Willemsen-Swinkels et al., 2000), we hypothesized that, compared to children classified as organized insecure or disorganized, children classified as securely attached would be more likely to initiate social interactions with their caregiver and would be more responsive to their caregiver’s bids for play.

## Method

### Participants

The participants in this study were recruited through the UCLA Autism Evaluation Clinic between 1997 and 2000. Clinic staff contacted parents of young children with a clinical diagnosis of autism spectrum disorder, informed them about the study, and provided them with a contact phone number if they wished to participate. Forty families were enrolled in the study, and 30 were seen in the Ainsworth Strange Situation Procedure (Ainsworth & Wittig, 1969). Children’s clinical diagnoses were confirmed using the Autism Diagnostic Interview-Revised (ADI-R; Lord, Rutter, & Le Couteur, 1994) and the Autism Diagnostic Observation Schedule-Generic (ADOS-G; Lord, Rutter, & DiLavore, 1998). The sample was composed of 23 boys and 7 girls, with a mean chronological age of 47 months (SD = 9). Children entering the study had an average nonverbal mental age (NVMA) of 31 months (SD = 14) and an average overall language age of 21 months (SD = 13). At the 1-year follow-up, the sample’s average NVMA was 40 months (SD = 21) and average overall language age was 27 months (SD = 17). Hence, this was a heterogeneous sample in terms of children’s level of functioning. Twenty-eight children met the criteria for autism on either the ADOS or ADI, and the remaining two met the criteria for PDD-NOS on the ADOS-G; these children, originally assessed in the early 2000s, would today likely be subsumed under the Autism Spectrum Disorder diagnostic category in DSM-5.

### Procedures

The initial assessment occurred during two laboratory sessions at the UCLA Medical Center and included administration of measures of language and cognitive ability, as well as an assessment of empathy. In addition, during the initial assessment, children and their primary caregiver participated in Ainsworth’s Strange Situation Procedure (Ainsworth et al., 1978; Ainsworth & Wittig, 1969). A third assessment session was conducted in the families’ homes, where child–caregiver interactions were videotaped. Children were seen for follow-up assessments approximately 1 year following the initial laboratory visit (mean interval = 12.3 months, SD = 1.5 months), at which time all the assessments except for the strange situation were re-administered.

#### Strange Situation Procedure

Children and their primary caregivers (all mothers except, in one case, the father) were seen in the Strange Situation Procedure (Ainsworth et al., 1978; Ainsworth & Wittig, 1969). Briefly, this procedure consists of eight standard episodes of increasing (moderate) stress during which the child is exposed to an unfamiliar room and a stranger, both in the caregiver’s (hereafter, parent) presence and in her absence, and undergoes two separations from and two reunions with the parent. During the first separation, the child is left in the presence of the stranger, and during the second separation, the child is left in the room alone. Separation episodes are terminated early if the child becomes unduly distressed. Classification of a child’s security with the parent is based on the child’s reaction to both reunions with the parent, rated with scales for proximity seeking, contact maintenance, avoidance, and resistance. The strange situations were videotaped with cameras mounted in three corners of the playroom.

Using these videotapes, Erik Hesse and Mary Main classified the children’s attachment organization using Ainsworth’s system (Ainsworth et al., 1978), the infant disorganized/disoriented classification (Main & Solomon, 1990), and the 6-year old system for coding (formerly disorganized) children as controlling (Main & Cassidy, 1988). Hesse and Main had had previous experience classifying attachment in children with autism (see Capps et al., 1994). Both coded each child’s strange situation independently; three disagreements were resolved through discussion. As Naber et al. (2007b) noted in conjunction with their own study, when coding disorganization in children with autism, observers should take the individual’s own baseline behavior – as displayed in the first few strange situation episodes – into account, to differentiate between conventionally identified disorganized/disoriented behavior and those anomalous behaviors that are typical for children with autism. A further criterion for assessing the disorganization of attachment, following Pipp-Siegel and colleagues (1999), was whether these behaviors were shown in connection with the mother (upon separation and reunion) or indiscriminately throughout the session. In addition, certain behaviors (not in fact listed among Main and Solomon’s 1990 indices of disorganization) were more likely to be excluded as expectable of children with autism, such as stereotypic hand flapping, squealing, or perseverative treatment of objects. Other disorganized behaviors, however, appeared to have a relational basis, such as simultaneous approach/avoidance (e.g. moving toward the parent with head averted or briefly lying on the floor with the forehead down on the arm immediately upon parent’s reentry to the room). Hesse and Main therefore focused on those aspects of the Main and Solomon system as to distinguish (1) children whose behavior was more readily explicable in terms of autism (D in the context of autism, or “D-Autism” for short) from (2) children who showed behavior more readily explicable in terms of (ordinarily brief) breakdowns or disruptions in the child’s attachment system (“D-Attachment”). This procedure was undertaken in the hopes of creating a more sensitive behavior-by-behavior differentiation of disorganization from autism than studies that only focused on excluding stereotypic behaviors from consideration (e.g. Willemsen-Swinkels et al., 2000). In this study, some stereotypic behaviors did contribute to a D-Attachment score, for instance, when a stereotypic behavior – such as hand flapping – only occurred on reunion and substantially interrupted the child’s approach. In contrast, behaviors commonly observed in children with autism, such as twirling and tip toe walking, while fitting in principle to the D headings, had not been observed by these coders in any neurologically normal children.

#### Parent–child interaction

Parents were told that we wanted to observe them interacting with their children as they typically would during play. Parent–child interactions were videotaped during a separate visit to the families’ homes. Parents were asked to play with their children using the child’s own toys (unstructured play, 15 min), as well as with a standard set of toys (puzzles, blocks, cars, balls, books, string, a shape-sorter, a pop-n-pal, a jack-in-the-box, tea set, doll) provided by our laboratory (structured play, 15 min).

Ratings of parental sensitivity were undertaken across the 30 min of play interaction by two independents observers, blind to the child’s attachment classification, and using Ainsworth’s original sensitivity–insensitivity scale (Ainsworth, Bell, & Stayton, 1974; Ainsworth et al., 1978). The interclass correlation based on videotapes of 10 mother–child interactions was .83. Additionally, the Mother–Child Rating Scale developed by Crawley and Spiker (1982) was used to rate the quality of the children’s social initiations and social responsiveness, both rated on a 5-point Likert scale. Both verbal behaviors (vocalization and language use) and nonverbal behaviors (conventional gestures and looks) were considered in assigning the ratings for quality of initiations and responses. The assigned *social initiative* rating reflected the degree to which the child initiated interactions with the mother, with frequency, intensity, and variety of initiations taken into consideration when assigning the rating. Initiating behaviors included simply looking at the mother, looking and vocalizing (or talking), looking and pointing, offering objects to the mother, and pulling on the mother to get her to do something. The assigned *social responsivity* rating reflected the degree to which the child positively responded to maternal initiations and included frequency, duration, and latency of response. The interclass correlations for inter-rater reliability on the two ratings, based on videotapes of 10 independently coded parent–child interactions, were .91 for *social initiative* and .89 for the *social responsivity*.

### Assessments of cognitive skills

Children’s nonverbal cognitive abilities were assessed using two subscales of the Mullen Scales of Early Learning (MSEL; Mullen, 1995). These were the Visual Reception Scale and the fine motor scale. Both scales generate age equivalents, which were then averaged to generate a NVMA. One child passed the highest items on the MSEL, and for that child, the NVMA score was based on two nonverbal subscales of the Stanford–Binet Intelligence Scale (Pattern Analysis and Bead Memory; Thorndike, 1972).

The current sample was heterogeneous with respect to children’s language abilities, which precluded using a single standardized measure of language. Consequently, three different tests were administered, based on the child’s functional level: (1) Reynell Developmental Language Scales (RDLS; Reynell, 1983), (2) Childhood Evaluation of Language Fundamentals – Revised (CELF-R; Semel, Wiig, & Secord, 1987), or (3) MSEL (Mullen, 1995; Expressive and Receptive Language Scales). At the first time point, 18 (60%) of the language evaluations were based on the RDLS. Twelve children did not obtain the basal score on the RDLS necessary to proceed with the assessment and were thus given the MSEL (40%). At the second time point, 23 (76%) of the children were administered the RDLS, 5 (17%) were given the Mullen, and 2 (7%) obtained a basal score on the CELF-R. These measures provide age equivalents for children’s receptive and expressive language skills, and the average of children’s receptive and expressive age equivalents was used to compute an overall language age equivalent.

#### Assessment of empathy

Children’s responsiveness to the examiner’s display of apparent distress was observed using a structured empathy task (see e.g. Dissanayake & Sigman, 2000; Hutman et al., 2010; Sigman et al., 1992). One child participated in the empathy assessment at the follow-up visit only; all other children participated in the assessment during both the intake and follow-up visits. The child and the examiner are seated at a small table, and the examiner, while playing with a plastic pounding toy and a small hammer, pretends to hurt her finger by hitting it with the hammer. The examiner then displays vocal and facial expression of distress for 30 s, followed by 10 s of neutral affect. After that 10 s, the examiner reassured the child that she was “all better.” The procedure was videotaped, and both the child’s behaviors toward the examiner as well as the quality and intensity of the examiner’s distress display were scored. The child’s degree of interest and concern was coded using a 6-point rating scale: (1) shows no interest, (2) shows a hint of interest, (3) shows some apparent interest but no clear sign of concern, (4) shows one sign of concern, (5) shows more than one clear sign of concern, and (6) shows intense affective involvement and/or comforting behavior.

Based on videotapes of 10 children, inter-rater reliability for the coding of the participants’ empathy and the examiner’s intensity and quality of display of affect was established between consensus scores of two observers with a third independent observer. The interclass correlation for ratings of children’s empathy was .86, and for the examiner’s intensity and quality of affective display was 1.00. In all cases, the examiners’ affective display during the feigned distress received the maximum score for both intensity and quality.

## Results

### Attachment organization

All the children in this study showed clear indications of attachment to their parent in the Strange Situation. As usual in coding strange situations, the sample was first classified in terms of Ainsworth’s secure (B), avoidant (A), and resistant (C) patterns, with ambiguous cases being forced into the best-fit Ainsworth category. This resulted in 17 children being placed in the secure group and 12 being placed in the insecure group. Then, using the Main and Solomon indices for disorganized/disoriented attachment, 7 children were classified as primarily secure (B), 5 children were classified as relationally disorganized (D-Attachment), and 17 children were classified D-Autism. One child was coded as “cannot classify” because he displayed a mixture of avoidant, resistant, and disorganized behaviors. Following protocol for such cases (Main & Hesse, 1992), this child was included in the insecure group with the children classified as organized insecure and as disorganized.

Of the five children who received a primary classification of D-Attachment, three were secondarily classified as secure (B), one as resistant (C), and one as avoidant (A). Of the 17 children who showed disorganized/disoriented behaviors that appeared readily attributable to autism (D-Autism), six were subclassified as secure (B), one as resistant (C), and five as avoidant (A). Four of these children showed disorganized/disoriented behavior which appeared attributable to autism but also had some features of relationally based disorganized/disoriented behavior. These four children were therefore classified as D-Autism with a secondary classification of D-Attachment. One child was dropped from the analysis, as the coders did not feel confident in making a judgment regarding whether the child’s behavior was more readily attributable to autism or relationally based disorganization.

As anticipated, the small sample size precluded three-way (ABC) and four-way (ABCD) analyses, so children were divided into two groups: secure and organized insecure/disorganized. The group classified as secure was composed of those showing no indices of D (*n* = 7) and those showing D-Autism with a secondary patterning of B (*n* = 6). Preliminary analyses indicated no differences between these two sets of children classified as secure on any of the dependent measures of interest. The second group comprised children with a primary classification of D-Attachment (*n* = 5), D-Autism with secondary A, C, or D (*n* = 10), and one child who displayed a blend of insecure attachment strategies together with disorganization. Based on these classification criteria, the sample consisted of 13 secure (45%) and 16 insecure (55%) children.

### Cognitive correlates of individual differences in attachment security

Four independent samples *t*-tests were conducted to compare children with secure and insecure classifications with respect to NVMA and language skills (calculated as an average of expressive and receptive language age) at intake and follow-up. To reduce the risk of Type 1 error associated with multiple comparisons, the Bonferroni adjustment was applied, with an adjusted alpha level of .013. The means and standard deviations for the language variables and NVMA for the secure and insecure groups can be found in Table 1. During the initial assessment, children with a secure attachment classification exhibited significantly higher language skills, *t*(27) = 3.45, *p* = .005, than children with an insecure classification, and these group differences persisted through the 1-year follow-up, *t*(27) = 3.19, *p* = .008. Using an adjusted alpha level of .013, the two groups did not differ with regards to NVMA at intake, *t*(27) = 2.76, *p* = .02, or at the 1-year follow-up, *t*(27) = 1.85, *p* = .08. However, a trend is evident in the results, and the secure children at initial assessment had a higher NVMA than the other infants even at the 1 year follow-up.

**Table 1.** Attachment security and children’s nonverbal and language abilities.

	Intake		One-year follow-up	
Child characteristics	Secure	Insecure	Secure	Insecure
Nonverbal mental age	37.7 (16.4)	24.8 (7.8)	47.9 (21.8)	34.2 (18.2)
Language age	28.9 (14.3)**	14.8 (7.2)	36.8 (19.0)**	19.5 (9.4)

### Parent–child interaction

Children in the securely and insecurely attached groups differed in terms of their language abilities, and maternal sensitivity itself was moderately and significantly correlated with children’s language abilities, *r*(29) = .42, *p* = .02. Therefore, the association between sensitivity and attachment classification was analyzed with an ANCOVA, with children’s overall language abilities entered as the covariate. As noted above, prior to coding disorganization, a best-fitting Ainsworth classification was assigned to each case. Using this Ainsworth secure versus insecure division, and after adjustment for language abilities, maternal sensitivity ratings for the securely attached group were significantly higher than maternal sensitivity ratings for the insecurely attached group, *F*(1,26) = 12.15, *p* = .002. By contrast, when disorganization was taken into account and children classified as secure were compared to children classified as organized insecure or as disorganized, caregiver sensitivity did not differ between groups, *F*(1,26) = 3.67, *p* = .12. Means and standard errors of the maternal sensitivity ratings can be found in Table 2.

**Table 2.** Mean ratings of maternal sensitivity and child sociability by attachment group.

	Unadjusted means^a^		Adjusted means^b^	
Mother/Child ratings	Secure	Insecure	Secure	Insecure
Maternal sensitivity^c^	6.9 (1.5)*	5.4 (1.5)	6.7 (.5)	5.6 (.4)
Maternal sensitivity^d^	7.0 (1.4)***	4.8 (1.0)	7.0 (.3)**	4.9 (.4)
Child social initiative	3.7 (.5)***	2.3 (1.0)	3.5 (.2)**	2.4 (.2)
Child social responsivity	4.4 (.8)***	2.9 (.9)	4.2 (.2)**	3.1 (.2)

One-way between-groups analyses of covariance were conducted to examine group differences between the secure children and others in terms of their initiations and responsiveness to their parent, again while controlling for children’s language abilities. In line with our hypotheses, after adjusting for initial language skill, securely attached children were significantly more likely than the others to initiate interactions with their parent, *F*(1,26) = 9.01, *p* = .006, partial eta squared = .26. Securely attached children were also significantly more responsive to the parents’ social initiations, *F*(1,26) = 9.35, *p* = .005, partial eta squared = .27. Means and standard errors of the child ratings are reported in Table 2.

### Attachment organization and children’s empathy

**Figure 1.**
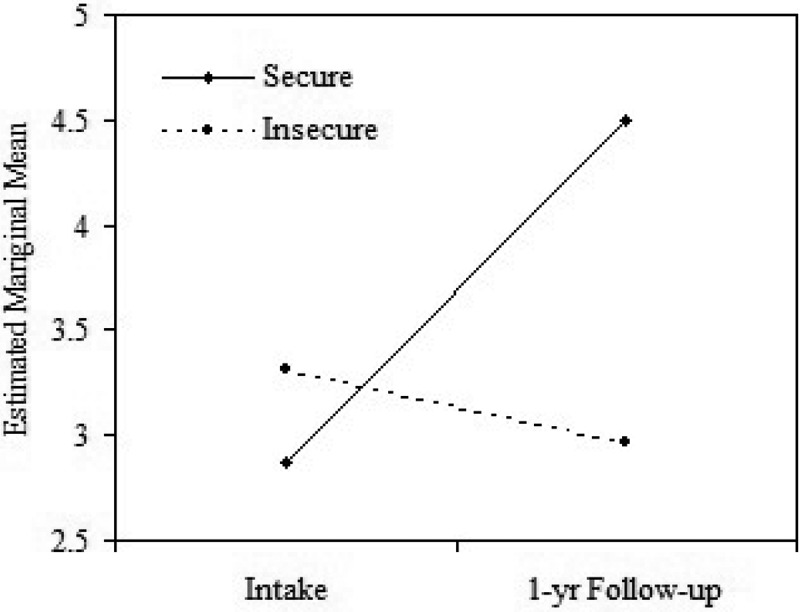
Mean empathy ratings at intake and follow-up for the secure and insecure groups (rated on a scale of 1–6). Means plotted are estimated marginal means, adjusted for children’s initial language ability.

Children’s empathy ratings at 1-year follow-up were significantly and highly correlated with their empathy ratings at study intake, *r*(29) *=* .59, *p* = .001, and with their initial language abilities, *r*(29) = .64, *p* = .001. Hence, to examine the *unique* contribution of attachment security to children’s empathy scores during the follow-up, the data were analyzed using a one-way repeated measures ANCOVA, with time (intake, follow-up) as a within-subject factor, secure attachment versus others (insecure + disorganized) as a between-subjects factor, and initial language skill as the covariate. The ANCOVA yielded a significant time by group interaction, *F*(1,25) = 8.88, *p* = .006, partial *η* ^2^ = .26 (see Figure 1). An analysis of simple main effects indicated that the empathy ratings for the securely attached group at the 1-year follow-up were significantly higher than their ratings at study intake, *F*(1,25) = 13.07, *p* = .001, whereas there was no change in empathy ratings from intake to follow-up for the other children, *F*(1,25) = .70, *p* = .41. Tests of within subject effects indicated that the main effect for attachment security was nonsignificant, *F*(1,25) = 1.06, *p* = .31, and the main effect for language was significant, *F*(1,25) = 14.76, *p* = .001. Thus, while language was a stronger predictor of an overall empathy score (aggregated across intake and follow-up) than attachment security, the effect of security on change in empathy from intake to follow-up was significant above and beyond the contribution of language.

## Discussion

The current study provides the first evidence that the sequelae of attachment security in autism appear to be similar to those reported for typically developing children. Controlling for initial language abilities, we found that children classified as securely attached were judged as more empathic to the apparent distress of another in an experimental context during a 1-year follow-up than other children whose attachments were identified as organized insecure or disorganized. We further confirmed that, controlling for children’s language skills and excluding disorganization from consideration, maternal sensitivity was associated with attachment security, replicating previous findings in autism (Capps et al., 1994; Koren-Karie et al., 2009; Oppenheim et al., 2012; see also Ainsworth et al., 1978). Finally, children classified as securely attached had higher language abilities (concurrently as well as during the 1-year follow-up) and directed more initiations to their caregivers and were more responsive to their bids during a separate play interactions conducted in the home.

Associations between autistic children’s language and social skills on the one hand and attachment security and maternal sensitivity on the other raise the issue of whether our measurement of these attachment-relevant constructs lacks discriminant validity in this population (Atkinson et al., 1999). With such concurrent associations, it could be argued that our measurement tools for evaluating attachment security and maternal sensitivity – via strange situation coding and maternal sensitivity ratings – are simply capturing level of functioning in ASD. One way to evaluate whether attachment classifications are valid is to examine whether they have the same developmental sequelae for children with autism as has been found in normative samples. Attachment theory posits that securely attached infants, who have their own emotional needs satisfied through consistent and sensitive caregiving in times of distress, may consequently be better attuned to the emotional needs of others and more capable of empathic responding. In line with these predictions as well as research with typically developing children (e.g. Kestenbaum et al., 1989; Sroufe, 1983), securely attached children in the current sample were judged to be more empathic to the examiner’s apparent distress during the 1-year follow-up visit relative to their empathy ratings at study intake. In contrast, children classified as insecure did not demonstrate gains in their empathy scores over the same period. Importantly, attachment security was linked to gains in empathy in the secure group above and beyond the contribution of language. By documenting a link between quality of attachment and subsequent empathic responsiveness of children with autism, the current study is the first to demonstrate that the consequences of secure attachment for children with autism may be similar to those reported for typically developing children (see e.g. Main & Weston, 1981).

Although these findings will need to be replicated in natural settings (e.g. by examining children’s responses to peers’ distress on the playground), and with larger samples, they nonetheless suggest that attachment security may be an important predictive variable to incorporate in our models of socio-emotional development in autism. Should future research confirm that attachment security predicts emotional responsiveness in autism beyond the contribution of other variables – language, nonverbal communication, representational ability, social attention – the quality of attachment may represent an important target in parent-based interventions for young children with autism (e.g. see Dolev, Oppenheim, Koren-Karie, & Yirmiya, 2014).

In terms of the goal of exploring the dynamics that underlie individual differences in attachment organization among children with autism, we found associations between attachment classification and children’s and parents’ behavior in a separate social interaction. Controlling for language ability, we found that the parents of children classified as securely attached were not significantly more sensitive than parents of children classified as organized insecure and disorganized, though the trend was in the expectable direction. By contrast, however, once we excluded disorganization (i.e. primary D codings) from consideration for this specific analysis, parents of children classified as securely attached *were* found to be significantly more sensitive than parents of children classified as insecure. We note that this may help make sense of the disagreement in findings between Capps and colleagues (1994) and Oppenheim and colleagues (2012) on the one hand and van IJzendoorn and colleagues (2007) on the other. We are persuaded by the explanation put forward by Van IJzendoorn et al. (2007) that, because their sample included infants rather than preschoolers, the sensitivity–security link seen in neurologically typical infants of the same age had yet to emerge within this developmentally younger sample of children with autism. This link requires more cognitive-developmental models of expectable child–parent interactions and intentions, which may well be a later achievement for children with autism. To this, we would add an additional possibility that the link between sensitivity and security was obscured in the findings of Van IJzendoorn et al. (2007) because 73% of their insecure group was composed of infants classified as disorganized. In contrast, only 33% of the insecure group in the study of Capps et al. (1994) and 38% of the insecure group in the study of Oppenheim et al. (2012) were composed of infants classified as disorganized. Since, from a theoretical perspective, you would not expect an association between caregiver sensitivity and disorganization (Out et al., 2009), the lack of association between maternal sensitivity and attachment in the study of Van IJzendoorn et al. (2007) may be due to the unusually high proportion of disorganized children in their sample.

Our findings indicate that maternal sensitivity may promote attachment security in children with autism in a way consistent with attachment theory. In addition, the impact of the developmental level of autistic children on the quality of parent–child interaction has been highlighted. In the current sample, parental sensitivity was moderately correlated with children’s language abilities. In addition, the secure children more frequently initiated social interactions with their parents and were more responsive to their parents’ social initiatives than the organized insecure and disorganized children. As Capps and colleagues (1994) had suggested, associations between attachment security, maternal sensitivity, and children’s language and social abilities raise a question about the direction of effects. On the one hand, as has been demonstrated for typically developing children, children with autism who experience sensitive parenting in the absence of frightened/frightening/dissociative behaviors outlined by Hesse and Main (2006) may be more likely to form secure attachments, which in turn support the development of their social and language skills. Indeed, Siller and Sigman (2002, 2008) have already documented a predictive relationship between responsive behavior – specifically, the degree to which mothers are able to synchronize their vocal behavior with their child’s focus of attention – and growth in language in children with autism. On the other hand, the level of socio-emotional and cognitive functioning in children with autism may affect their caregiver’s ability to perceive and read their signals and thus constrain the parent’s ability to respond consistently to these signals. *Autism disrupts the child’s ability and motivation to signal his or her needs effectively, and caregiver sensitivity must be seen within the context of these communicative limitations*. This fits with the finding by Van IJzendoorn et al. (2007) that severity of autistic symptoms was an important predictor of whether a child in their sample would be classified as secure or as either insecure or disorganized.

The associations between the child’s language level and maternal sensitivity ratings in the present sample may also be seen in light of wider findings that the child’s level of language impairment, rather than presence of autism in general, affects mothers’ behaviors toward their children with autism. Several studies have documented that mothers of children with autism use more directives and approach behaviors than mothers of typically developing children, particularly when interacting with less communicatively able children, and that these adjustments are effective in increasing children’s responsiveness and engagement (e.g. Kasari, Sigman, Mundy, Yirmiya, 1988; Nassan El-Ghoroury & Romanczyk, 1999; Doussard-Roosevelt, Joe, Bazhenova, & Porges, 2003). These maternal behaviors that increase the responsiveness of children with autism, such as physically holding the child on task (Kasari et al., 1988) or more explicitly directing the child’s behavior to elicit a response (Doussard-Roosevelt et al., 2003), may appear intrusive and do not reflect behaviors that are typically considered as evidence of sensitive parenting, such as following the child’s lead. Given that the primary caregivers of minimally verbal children with autism may be more directive than those of verbal children, it is possible that these caregivers may also be rated as less sensitive. Future research on maternal sensitivity in autism should consider how caregiver behaviors that elicit engagement from children with autism relate to our conceptualization of sensitive parenting. Sensitive parenting necessarily involves a caregiver being able to adjust to their child’s limitations, and the structure that supports engagement in children with autism (or typically developing children, for that matter) needs to be provided in a sensitive manner in order to be effective (Dolev, Oppenheim, Koren-Karie, & Yirmiya, 2009).

Relevant to the issue of the concomitants of attachment security in autism, we also found that securely attached children in our sample had superior language abilities compared to the group of organized insecure and disorganized children, concurrently as well as during a 1-year follow-up. Thus, our findings replicate and extend a substantial body of empirical evidence linking cognitive skill and attachment security in children with autism (Capps et al., 1994; Rogers et al., 1993, 1991; Willemsen-Swinkels et al., 2000), Down syndrome (Atkinson et al., 1999), and typically developing children (Main, 1983; Van IJzendoorn et al., 1995) and document these links longitudinally.

With respect to attachment organization, and in line with findings in the wider field (Rutgers et al., 2004), 45% of the current sample of children with autism were classified as secure, either as a primary classification or in conjunction with behaviors in the Main and Solomon indices which were readily explicable as stemming from autism rather than a relationally based disorganization of the attachment system. However, several children in the current sample showed disorganized behaviors that were not readily explicable in terms of autism – for instance, in being expressed specifically in connection with the attachment figure rather than indiscriminately throughout the session. Hence, we made a distinction between disorganized behaviors attributable to autism (D-Autism) from those behavioral indices of attachment disorganization stemming from the relationship, as they have been defined among typically developing children (D-Attachment). Further research is clearly needed to validate this distinction via assessment against expectable external correlates of disorganization, such as frightened and/or frightening parental behavior as described by Hesse and Main (2006) and parental withdrawing and role-confused behaviors as described by Lyons-Ruth and Spielman (2004). A greater understanding of the biobehavioral correlates of attachment disorganization in autism, such as heart rate variability (Willemsen-Swinkels et al., 2000) or cortisol levels (Luijk et al., 2010), may be especially helpful in distinguishing behaviors attributable to autism from those which, to varying degrees, indicate breakdown or disruption in the child’s attachment system. Researchers may also wish to examine attachment in the home setting and to interview the parent (or other primary caregiver) to obtain more specific information regarding which behaviors may be attachment related for the child with autism and which may be behaviors exhibited in the child’s general functioning.

A limitation of the present study stems from the fact that caregiver sensitivity and attachment security were assessed concurrently, precluding causal inferences. We cannot yet, then, establish sensitivity as an *antecedent* of security in this population, as has been demonstrated for typically developing infants (Ainsworth et al., 1978). A longitudinal study of infants at risk for autism (by virtue of having an older sibling with the condition), followed from the early months of life through an age when children’s diagnosis of autism can definitely be determined, is needed (Rogers, 2009). Such a design would enable the field to disentangle the relative contributions of caregiver behaviors and child characteristics to the development of attachment security in children with autism. Furthermore, longer term follow-up is needed to further establish the causal role of attachment security in the *development* of language, empathy, and other aspects of socio-emotional functioning in this group. A second limitation of our study is that our modest sample size precluded the possibility of examining correlates and consequences of individual differences in attachment security at finer resolutions than just the secure/insecure split. Studies with larger samples will be needed to address these critical research questions.
